# Posteromedial varus fatigue fragment (PVFF) in severe varus knee osteoarthritis phenotype: incidence, surgical implications, and management

**DOI:** 10.1051/sicotj/2025038

**Published:** 2025-07-23

**Authors:** Vaibhav Bagaria, Anjali Tiwari

**Affiliations:** 1 Director and Consultant, Department of Orthopedic Surgery, Sir H N Reliance Foundation Hospital Girgaum Mumbai 400004 Maharashtra India; 2 Research Analyst, Department of Orthopedic Surgery, Sir H N Reliance Foundation Hospital Girgaum Mumbai 400004 Maharashtra India

**Keywords:** Varus knee, Stress fracture, Posteromedial tibial plateau, ACL deficiency, Total knee arthroplasty, Tibial fixation, Medial gap balancing

## Abstract

*Purpose*: Severe varus knee osteoarthritis (OA) alters weight-bearing mechanics, leading to progressive stress concentration on the posteromedial tibial plateau. In select cases, this results in the development of a Posteromedial Varus Fatigue Fragment (PVFF), a chronic stress-related fracture that remains ununited and influences knee stability, surgical planning, and implant selection. This study aims to evaluate the incidence, radiographic detectability, and intraoperative significance of PVFF in patients undergoing total knee arthroplasty (TKA). *Methods*: A retrospective analysis was conducted of 856 consecutive TKA cases performed by a single surgeon. Preoperative radiographs, intraoperative findings, and surgical modifications were assessed to determine the incidence and implications of PVFF. Correlation with varus severity and absence of ACL was done. *Results*: PVFF was detected intraoperatively in 17 of 856 cases (1.99%), but only 9 (53%) were visible on pre-op imaging.” All PVFF cases exhibited varus alignment exceeding 15° and complete ACL deficiency. Intraoperatively, fragment removal resulted in an increased medial flexion gap, impacting gap balancing and necessitating adjustments in implant selection, including the use of tibial stems or augments in select cases. *Conclusion*: PVFF is an underrecognized structural lesion for precision in severe varus knee OA, affecting tibial fixation, load distribution, and medial knee stability. Its presence requires careful intraoperative assessment, as fragment removal can alter gap balancing. Improved preoperative recognition and surgical planning are essential to optimize TKA outcomes in patients. Further prospective studies and biomechanical analyses are needed to better understand PVFF’s long-term clinical implications and refine surgical strategies.

## List of Abbreviations

ACL: Anterior Cruciate Ligament
AP: Anteroposterior (radiographic view)
CT: Computed Tomography
HTO: High Tibial Osteotomy
KSS: Knee Society Score
MAD: Mechanical Axis Deviation
MRI: Magnetic Resonance Imaging
OA: Osteoarthritis
POL: Posterior Oblique Ligament
PVFF: Posteromedial Varus Fatigue Fragment
TKA: Total Knee Arthroplasty

## Introduction

Severe varus knee deformity is a well-recognized condition associated with advanced medial compartment osteoarthritis (OA) and altered knee biomechanics [1, 2]. As the knee progressively shifts into varus alignment, the mechanical load becomes disproportionately concentrated on the medial tibial plateau. Over time, this excessive and asymmetric loading can lead to structural compromise, including subchondral bone collapse, stress fractures, and progressive joint instability.

The anterior cruciate ligament (ACL) plays a critical role in maintaining knee stability by preventing excessive anterior translation of the tibia and resisting varus and rotational forces. However, in patients with long-standing severe varus deformity, the ACL often becomes functionally incompetent due to chronic laxity, attritional changes, or complete rupture [3]. Loss of ACL function, combined with varus malalignment, shifts the biomechanical load further posteromedially on the tibial plateau, increasing stress on the subchondral bone [4].

A unique but underrecognized consequence of this altered biomechanics is the development of chronic stress fractures in the posteromedial tibial plateau. Posteromedial varus fatigue fragment (PVFF) is a type of nonunion fatigue fracture, separate from classic tibial plateau fractures. Unlike acute traumatic fractures, these stress fractures develop insidiously due to repetitive microtrauma and bone fatigue. This results in the formation of a PVFF, which although separate from the main tibial plateau, retains its posteromedial attachments. Chronic stress fractures occur due to repetitive submaximal loading of the bone, which exceeds its capacity to repair and remodel. Bone is a dynamic tissue that remodels in response to mechanical stress through the activities of osteoclasts (bone resorption) and osteoblasts (bone formation) [5]. Typically, they result from an imbalance where bone resorption outpaces bone formation. Fatigue damage accumulates faster than the bone can remodel, leading to a fracture. The knee joint’s load distribution plays a critical role. In varus alignment, the increased load is placed on the medial compartment, while valgus alignment increases the load on the lateral compartment. This uneven load distribution can contribute to localized stress fractures [6].

Recognition of posteromedial tibial plateau stress fractures is crucial for surgical planning. In total knee arthroplasty (TKA), unaddressed stress fractures may predispose to implant failure or tibial collapse, necessitating augmented fixation techniques. Removal of these fragments detaches structures like the posterior oblique ligament (POL) and semimembranosus tendon. This results in a sudden medial gap opening, which complicates soft tissue balancing during TKA [7].

This study aims to investigate the incidence of posteromedial tibial plateau stress fractures in patients with severe varus knee deformity and evaluate their association with ACL incompetence. Additionally, we seek to analyze the clinical and surgical implications of these fractures in the context of managing advanced varus knee OA. While posteromedial tibial stress fractures remain underrecognized in this population, their presence may significantly influence treatment algorithms for joint realignment, arthroplasty, or ligament reconstruction. By systematically characterizing this phenomenon, our findings aim to refine preoperative diagnostic protocols, inform tailored surgical planning, and optimize outcomes for patients with severe varus malalignment. This work underscores the importance of identifying such fractures during the evaluation of degenerative varus knees, as their oversight may compromise the stability and longevity of corrective interventions.

## Materials and methods

### Study design

This single-center retrospective cohort study analyzed consecutive patients undergoing primary TKA performed by a single senior surgeon at a tertiary care orthopedic institution. The primary objective was to determine the incidence of posteromedial chronic stress fractures, identified radiographically by the presence of a PVFF, in patients with severe varus knee OA. Knee Society Score (KSS) was used to measure the functional outcome.

### Patient selection

#### Inclusion criteria

Patients were eligible if they underwent primary TKA for advanced varus knee OA and had standardized preoperative radiographic imaging (weight-bearing anteroposterior, lateral, and long-leg alignment views) available for retrospective review.

#### Exclusion criteria

Patients were excluded for prior tibial plateau or proximal tibial fractures, inflammatory arthritis (e.g., rheumatoid or psoriatic arthritis), metabolic bone disease (e.g., osteoporosis, Paget’s disease), secondary OA attributed to traumatic injury, revision TKA procedures, or prior high tibial osteotomy (HTO).

### Preoperative imaging evaluation

All patients underwent standardized weight-bearing knee radiographs, including:

1. Anteroposterior (AP) view: Assessed joint space narrowing and varus alignment.
2. Lateral view: Evaluated sagittal tibial plateau morphology.
3. Full-length hip-to-ankle standing radiographs: Measured mechanical axis deviation (MAD) and overall limb alignment.

### Diagnostic criteria for posteromedial varus fatigue fragment (PVFF) (Figure 1)

The fragment was identified preoperatively on standard radiographs if the following findings were observed:

1. Cortical irregularity or discontinuity with the presence of a distinct fragment in the posteromedial area at or below the joint line
  - A radiolucent line or subtle cortical break in the posteromedial tibial plateau.
  - Most evident in the lateral or Rosenberg views.
2. Subchondral sclerosis and bone reaction
  - Localized increased density or sclerosis in the posteromedial tibial rim.
  - Indicative of chronic stress-related bone remodeling.
3. Posteromedial subchondral depression
  - In some cases, early signs of collapse or depression of the posteromedial tibial plateau were observed.

**Figure 1:**
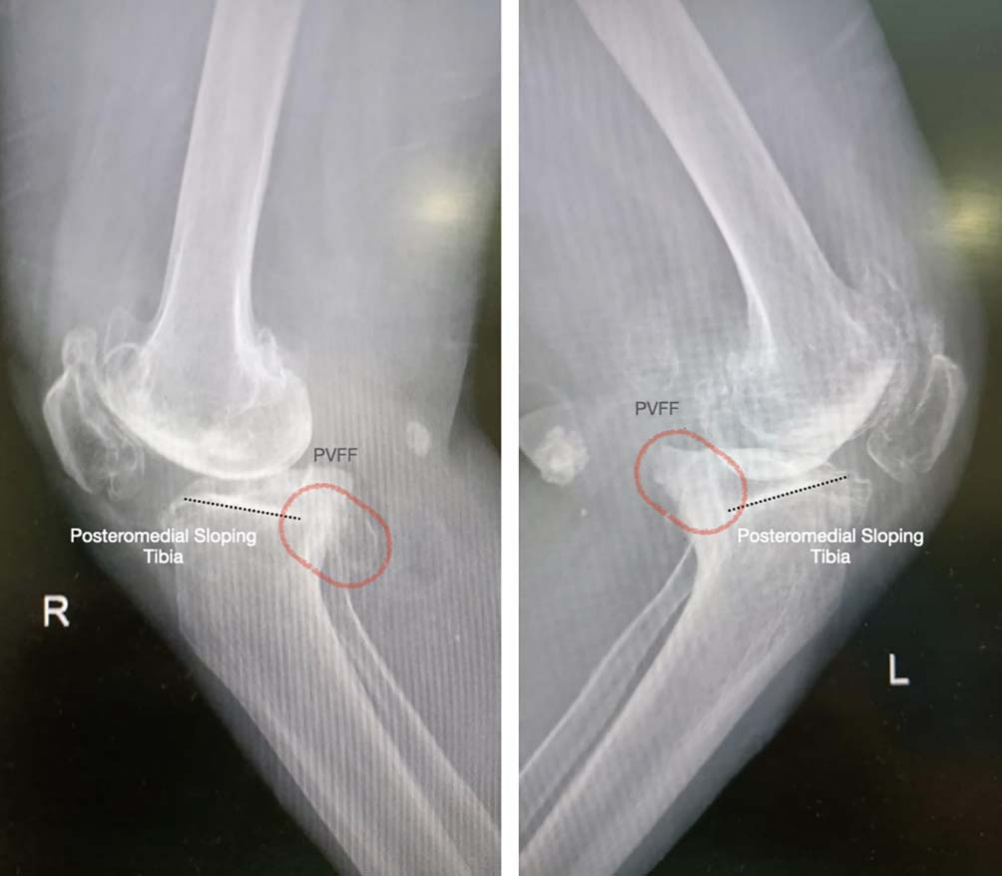
Illustration posteromedial fragment.

### Intraoperative identification of PVFF

**Figure 2:**
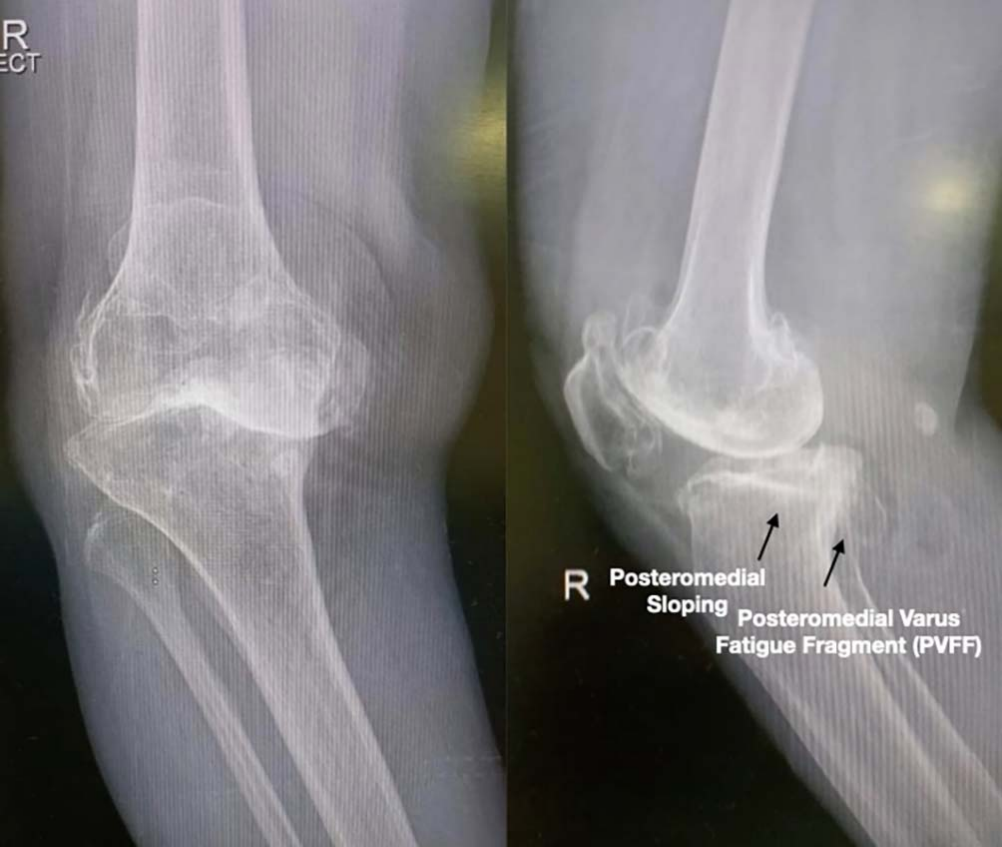
Preoperative radiographs showing varus deformity and posteromedial varus fatigue fragment (PVFF).

Surgical exposure and fragment visualization: During the standard medial parapatellar approach for TKA, meticulous soft tissue handling, and bone exposure allowed direct visualization of the posteromedial tibial plateau. The fragment was intraoperatively confirmed, presenting with the following characteristics (Figure 2):

1. Fragment morphology
  - The PVFF was located posteromedially along the tibial plateau rim.
  - It appeared as a thin cortical shell with subchondral bone involvement.
  - In some cases, the fragment was partially mobile or displaced, affecting the integrity of the tibial preparation.
2. Cortical disruption and bone quality
  - The posteromedial tibial rim showed signs of stress-related bone remodeling, with areas of bone resorption, sclerosis, or microfractures.
  - The quality of the remaining tibial bone was often compromised, necessitating additional fixation strategies.
3. ACL deficiency in all PVFF cases
  - In all 17 cases, the ACL was either completely absent or functionally incompetent, reinforcing the hypothesis that ACL deficiency contributes to increased posteromedial tibial stress.

### Surgical implications of PVFF

All the surgeries were performed by the same surgeon. The presence of the PVFF warranted a change in surgical technique, primarily aimed at balancing techniques and ensuring adequate coverage of the tibial defect. The choice of fragment retention and excision was based on intraoperative assessment. Typically, the fragments that prevented adequate gap balancing and required extensive releases were excised as the release left them avascular and potentially as a floating loose body.

1. Modification of tibial preparation: In cases with mobile or unstable fragments, careful removal or fixation of the posteromedial rim was required if the fragment was deemed fixable. Tibial cuts were adjusted to cover the excessive bone loss at the posteromedial aspect where feasible (maximum planned poly less than or equal to 13).
2. Use of augmented fixation strategies: Where indicated, additional screws, augments, or stems were used to reinforce the tibial component.

### Statistical analysis

Patient demographics, radiographic findings, and intraoperative observations were systematically documented. Categorical variables (e.g., sex, ACL status) are reported as frequencies and percentages, while continuous variables (e.g., age, varus angle) are expressed as means with ranges. The incidence of PVFF was calculated as the proportion of confirmed cases relative to the total cohort (*n* = 856). The sensitivity of preoperative radiographic detection was determined by comparing radiographically identified PVFF cases (*n* = 9) to intraoperatively confirmed cases (*n* = 17). Associations between PVFF, severe varus malalignment (>15°), and ACL incompetence were analyzed using Fisher’s exact test for categorical variables. Postoperative functional outcomes (Knee Society Score, KSS) were compared pre- and postoperatively using paired *t*-tests. A two-tailed *p*-value <0.05 was considered statistically significant. Statistical analyses were performed using SPSS v.26 (IBM Corp.).

## Results

### Incidence and demographics

Among 856 consecutive primary TKA cases, PVFF was intraoperatively confirmed in 17 patients (1.99% incidence). Preoperative radiographs identified PVFF in 9 of these 17 cases, yielding 52.9% sensitivity for radiographic detection. The cohort with PVFF comprised 5 males (29.4%) and 12 females (70.6%), with a mean age of 62.4 years (range: 54–78) (Table 1).

**Table 1:** Demographic, radiographic, and outcome characteristics of patients with posteromedial varus fatigue fragment (PVFF) undergoing total knee arthroplasty.

Parameter	Value	*p-*value
Total TKA cases	856	–
PVFF incidence	17 (1.99%; 95% CI: 1.2–3.2%)	–
Male: Female ratio (PVFF)	5:12	–
Mean age	62 years (range: 54–78)	–
Mean preoperative varus angle	17.2° (range: 15–39°)	–
Radiographic sensitivity	9/17 (52.9%; 95% CI: 29.7–75.2%)	–
ACL incompetence association	14/17 (82.4%; 95% CI: 59.0–93.8%)	0.003
Preoperative KSS	42.1 ± 8.3 (SD)	–
Postoperative KSS	86.7 ± 6.1 (SD)	<0.001
Follow-up duration	2 years (range: 6 months–5 years)	–

### Clinical and radiographic findings

**Figure 3:**
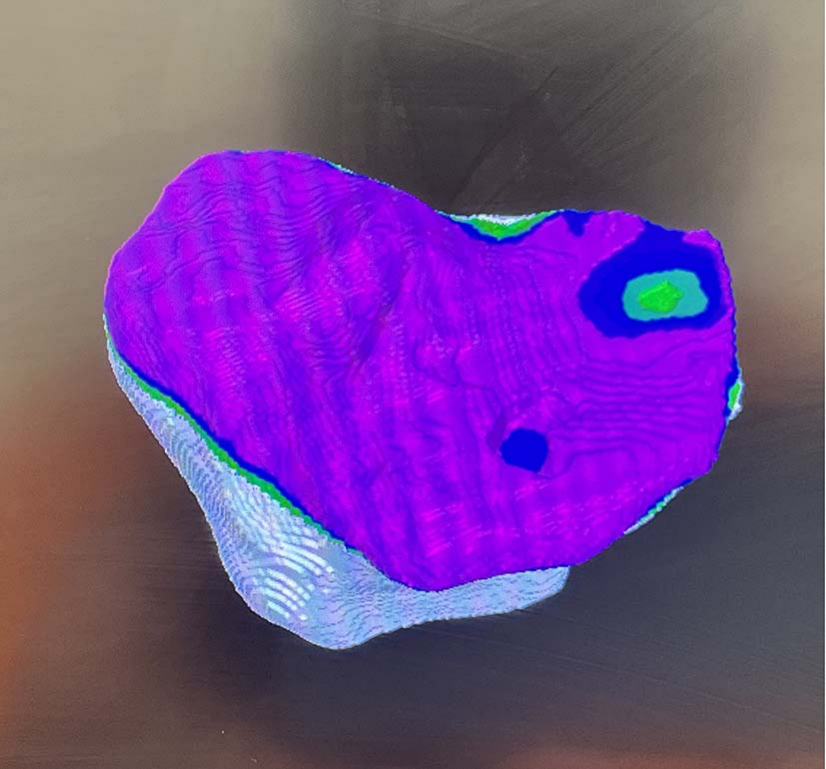
Image from robotic screen showing the defect on posteromedial corner of tibia.

**Figure 4:**
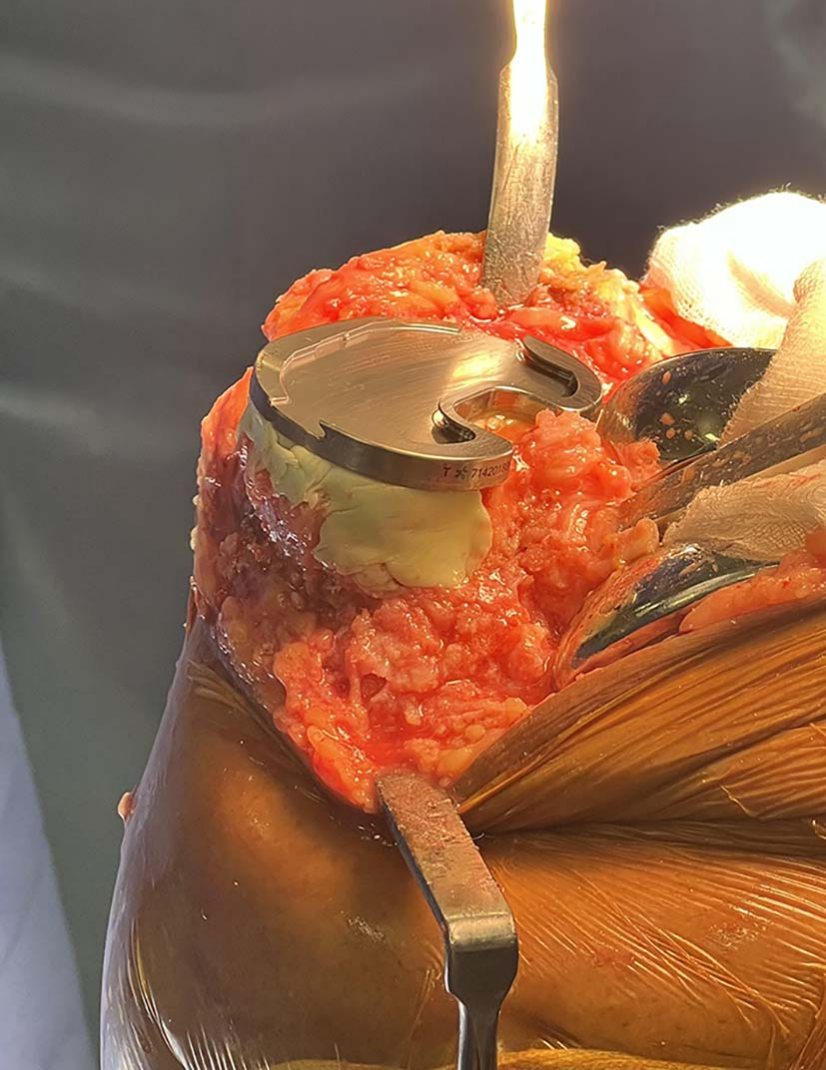
Alternative way of managing the defect with cement buildup.

All 17 patients exhibited severe varus malalignment, with a mean preoperative varus angle of 17.2° (range: 15–39°). Radiographic features of PVFF included posteromedial cortical discontinuity, subchondral sclerosis, and plateau depression. Preoperative imaging successfully identified PVFF in 9 cases (53%), while the remaining 8 cases (47%) were diagnosed intraoperatively. A moderate association was observed between PVFF and ACL incompetence, with 82.4% (14/17) of PVFF cases demonstrating ACL insufficiency (*p* = 0.003) (Figures 3 and 4). While there was no clear evidence on why some PVFF lesions were missed radiologically, it is postulated that it may have a temporal association with long-standing PVFF becoming sclerotic and more visible on X-rays and the acute fractures, especially non-displaced ones being missed.

### Surgical technique: intraoperative findings necessitated surgical modifications in all PVFF cases (Figures 5a and 5b)

All patients were monitored clinically and radiologically for any signs of loosening, radiolucent lines, or implant failure and migration.

**Figure 5:**
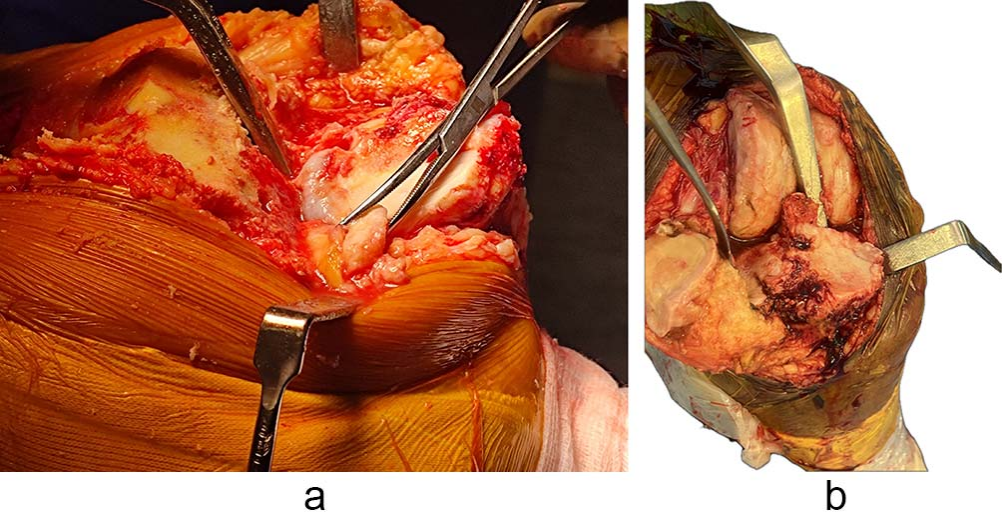
(a) Intraoperative images demonstrating the presence of PVFF. (b) Impending Fracture seen on posteromedial corner.

Fragment management: Unstable fragments were stabilized using compression screws (9/17) or excised (8/17) based on fragment size and bone quality.

Implant selection: Stemmed tibial components were utilized in 14 cases (82.4%) to bypass stress concentrations and bone grafting was performed in 5 cases (29.4%) with significant metaphyseal defects.

Ligament balancing: Increased medial soft-tissue releases were required due to chronic varus deformity and fragment-related instability.

### Postoperative outcomes (Figures 6 and 7)

At a mean follow-up of 2 years (range: 6 months–5 years), no cases of implant loosening, periprosthetic fracture, or revision surgery were reported. The mean KSS improved significantly from 42.1 ± 8.3 preoperatively to 86.7 ± 6.1 postoperatively (*p* < 0.001). The radiographic evaluation confirmed stable implants in all cases.

**Figure 6:**
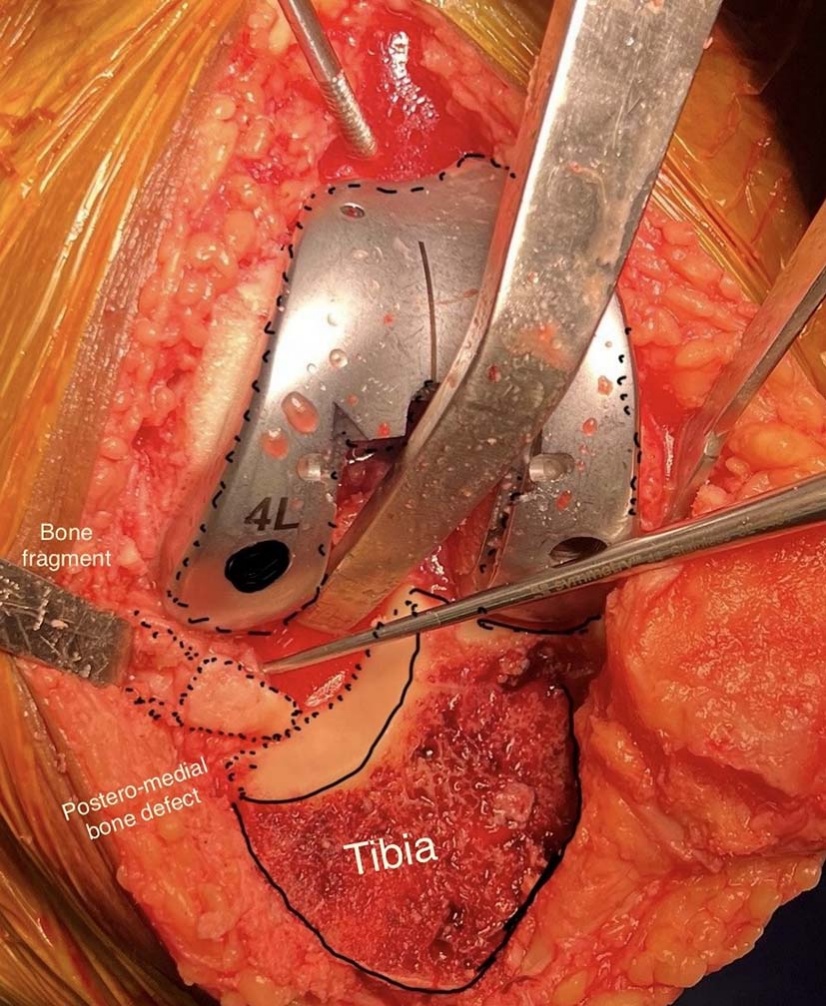
Intraoperative illustration of the posteromedial sloping, sclerosis, and fragment.

**Figure 7:**
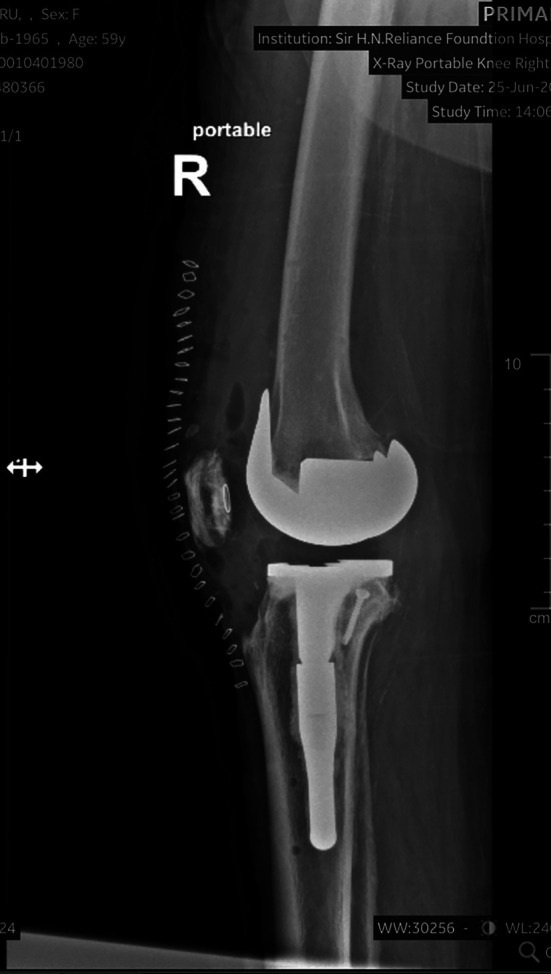
Postoperative radiographs showing defect managed with stem and screw.

## Discussion

The development of the PVFF in severe varus knee OA can be explained by the progressive biomechanical overload on the medial tibial compartment. In patients with significant varus deformity, the mechanical axis shifts medially, causing excessive concentration of load on the medial tibial plateau [8]. Normally, the weight-bearing axis of the knee passes slightly medial to the center of the joint, but in severe varus knees (>15° varus alignment), the axis shifts further medially, leading to abnormally high compressive forces on the medial compartment while simultaneously reducing load transmission through the lateral tibial plateau [9].

As a result of chronic overloading, the medial tibial plateau undergoes accelerated cartilage degeneration due to increased pressure and shear forces, making it progressively less effective at distributing loads. The subchondral bone responds to the persistent stress by becoming sclerotic and denser, a compensatory adaptation aimed at reinforcing the weakened joint surface. However, this sclerotic bone also loses its shock-absorbing capacity, leading to the transmission of higher loads directly into the posteromedial tibial subchondral region, where stress is concentrated due to varus malalignment. Over time, the inability of the subchondral bone to dissipate these forces effectively results in chronic microtrauma and fatigue failure, culminating in a stress fracture of the posteromedial tibial plateau [10, 11].

Once the posteromedial stress fracture occurs, the fragment often fails to unite, likely due to persistent mechanical overload, inadequate vascularity, and micromotion within the fragment. The presence of this unstable fragment may contribute to worsening instability in the knee, exacerbating varus thrust gait, which is a hallmark clinical feature of severe varus deformity [8, 9]. Varus thrust refers to the sudden lateral deviation of the knee during the stance phase of ambulation, indicating excessive dynamic loading on the medial compartment. Further gait analysis studies are required to delineate causality. This phenomenon suggests either a consequence or a contributor to PVFF, though biomechanical studies have yet to determine whether varus thrust is a precursor to stress fracture formation or a result of its presence.

Emerging finite element and gait analysis studies suggest that as medial compartment loading increases, the posteromedial corner of the tibial plateau may experience greater tensile and shear forces, particularly in ACL-deficient knees where the secondary stabilizing function of the ACL is lost. This further predisposes the region to chronic fatigue fractures, ultimately leading to the formation of PVFF [12].

Recognizing the PVFF intraoperatively is critical in optimizing knee replacement outcomes, as it directly impacts implant choice, tibial fixation, and soft tissue balancing. The altered weight-bearing pattern in severe varus knees creates localized posteromedial bone loss, which, if not accounted for, can compromise tibial baseplate coverage and lead to inadequate fixation. In cases where osteoporosis is present, the reduced bone stock further complicates implant stability, often necessitating the use of tibial stems or augments [13–15].

A key consideration during surgery is the role of PVFF in medial soft tissue stability. Despite being a separate fragment, it remains attached to critical stabilizers such as the posterior oblique ligament (POL) and semimembranosus tendon, which contribute significantly to medial knee balance. When the fragment is removed, there is often a sudden increase in the medial flexion gap, altering the expected balance and potentially resulting in excessive medial laxity. The surgeon must be prepared to fine-tune gap balancing intraoperatively, as failure to account for this change may lead to instability, necessitating constrained implants or thicker polyethylene inserts to restore stability. Uncontained defects and depression of more than 10 mm usually warrant the use of stems, augments, and in case of severe laxity, a constrained implant. This is based on intraoperative judgment by the surgeon.

Addressing PVFF requires a thoughtful surgical approach, particularly in determining whether to retain or excise the fragment. If it is stable and well-integrated, incorporating it into the tibial preparation may be preferable to preserve bone stock. However, if the fragment is nonunion or unstable, careful removal is necessary, with attention given to posteromedial tibial resection adjustments to maintain adequate fixation and prevent excessive bone loss. After fragment removal, reassessing gap balance and soft tissue tension is crucial, as intraoperative trialing may reveal unexpected medial laxity that must be compensated for with appropriate implant selection and soft tissue reinforcement [15].

Surgeons must remain vigilant during tibial preparation and trial reductions, particularly in cases where PVFF is present. The intraoperative plan must be flexible, with adjustments in implant choice, augmentation strategies, and balancing techniques dictated by the stability of the posteromedial compartment. A failure to recognize and address the implications of PVFF may lead to poor load distribution, tibial component loosening, and suboptimal long-term outcomes [15–17].

This study provides valuable insights into the presence and clinical significance of the PVFF in severe varus knee OA, but several limitations must be acknowledged. First, the study is retrospective, which inherently limits the ability to establish causality between varus deformity, ACL deficiency, and PVFF formation. While the intraoperative identification of PVFF was systematically documented, the preoperative detection rate was only 53%, suggesting that subtle cases may have been missed on standard radiographs, highlighting the need for advanced imaging modalities such as CT or MRI in select patients. Preoperative CT may be indicated in patients with varus >15° and suspected PVFF on radiographs.

Another limitation is the lack of long-term clinical follow-up to assess the impact of PVFF on postoperative outcomes. While intraoperative modifications were made based on the presence of the fragment, the effect on implant longevity, postoperative stability, and functional outcomes remains unknown. Prospective studies with longer follow-up periods would help determine whether specific implant strategies, such as tibial stems or augments. Augmented fixation (e.g., stems) may improve stability in compromised bone, though further evidence is needed.

Additionally, while the study identifies ACL deficiency as a consistent finding in all PVFF cases, it remains unclear whether PVFF formation is a direct consequence of ACL incompetence or an independent feature of advanced varus deformity. Biomechanical studies utilizing finite element analysis or cadaveric models would help clarify the sequence of mechanical failure that leads to PVFF formation and whether certain preventive measures, such as earlier intervention or bracing, could alter its progression [18].

Future research should also explore whether varus thrust gait contributes to or results from PVFF formation, as the relationship remains uncertain. Gait analysis and dynamic weight-bearing studies could provide deeper insights into how mechanical loading patterns influence stress fracture development and whether modifications in surgical technique, including soft tissue releases or alignment strategies, could mitigate its effects.

Given the growing recognition of PVFF, a standardized radiographic classification system would be beneficial to improve preoperative detection, guide surgical decision-making, and facilitate comparisons across future studies. A multi-center prospective study could help validate the clinical relevance of PVFF and refine management strategies to optimize outcomes in patients undergoing knee arthroplasty for severe varus deformity.

## Conclusion

In conclusion, the PVFF represents a distinct but underrecognized consequence of chronic mechanical overload in severe varus knee OA, particularly in the setting of ACL deficiency. PVFF alters intraoperative gap balance, necessitating anticipation of medial laxity and adaptive implant selection. Intraoperative identification is critical, as removal of the fragment can lead to unexpected medial laxity, necessitating modifications in gap balancing and implant choice. While this study highlights the importance of recognizing PVFF, further prospective research, biomechanical studies, and long-term outcome analyses are needed to refine preoperative detection and optimize surgical strategies for patients with this unique pathology.

## Limitations

This retrospective, single-center study has inherent selection bias, a low PVFF incidence limiting statistical power, and short-term follow-up. Interobserver variability in radiographic diagnosis was not assessed; differences in interpretation may influence preoperative detection rates. A notable limitation is the interobserver variability in radiographic diagnosis, with only 53% of PVFF cases identified preoperatively, reflecting challenges in detecting subtle signs (e.g., cortical breaks). The absence of routine CT/MRI may underestimate PVFF prevalence. Future work requires standardized imaging criteria and multi-center validation.

## Data Availability

The research data associated with this article are included within the article.
